# Differential attainment in public health specialty training recruitment in the United Kingdom: an observational analysis of applicants from 2018 to 2020

**DOI:** 10.1093/pubmed/fdac122

**Published:** 2022-11-03

**Authors:** Fran Bury, Mala Rao, Richard Pinder

**Affiliations:** Department of Primary Care and Public Health, School of Public Health, Imperial College London, London W6 8RP, UK; Department of Primary Care and Public Health, School of Public Health, Imperial College London, London W6 8RP, UK; Ethnicity and Health Unit, Department of Primary Care and Public Health, Imperial College London, London, W6 8RP, UK; Department of Primary Care and Public Health, School of Public Health, Imperial College London, London W6 8RP, UK

**Keywords:** differential attainment, ethnicity, medical training, recruitment, Public health

## Abstract

**Background:**

Differential attainment has been widely observed in United Kingdom (UK) medical training, with minority ethnicity being associated with reduced success in recruitment and progression through training. Specialty training in Public Health in the UK recruits candidates with medical as well as non-medical backgrounds. At the request of the UK Faculty of Public Health and Health Education England, we sought to examine whether differential attainment may or may not be occurring in the multi-stage recruitment process.

**Methods:**

We analysed 3 years of national recruitment data into Public Health specialty training to identify whether demographic characteristics including age, sex, ethnicity and professional background were associated with successful recruitment.

**Results:**

In total 2252 applications between 2018 and 2020 were analysed. Candidates who were older, Asian, black or from backgrounds other than medicine were significantly less likely to progress from the psychometric testing stage than the white British group. Fewer statistically significant differences were observed at the final stage of recruitment involving interviews, group work and a written task.

**Conclusions:**

The findings suggest that older candidates those from some ethnic minority backgrounds and those from backgrounds other than medicine are disadvantaged by the current recruitment process, with differential attainment associated with the psychometric testing stage.

## Introduction

Recruitment into Public Health specialty training in the United Kingdom (UK) is undertaken on an annual basis across all four countries of the UK. It is unusual among medical specialties, and internationally, in being open to candidates with a background other than medicine (BOTM) as well as qualified doctors. To develop a process which is fair for candidates from a diverse range of backgrounds, in 2009, a job analysis was undertaken, a person specification developed and a recruitment process designed. This process was evaluated and found to have good predictive validity for progression through training.^1^

**Fig. 1:**
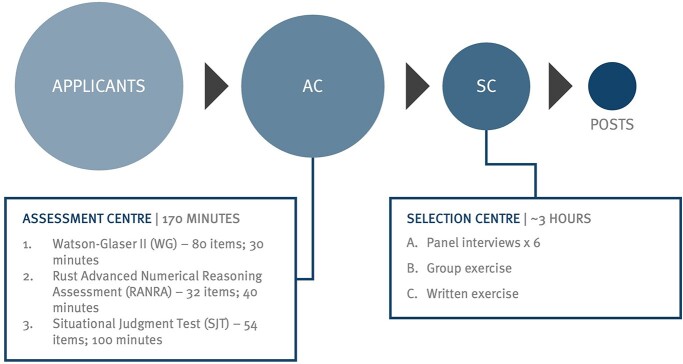
Recruitment cycle diagram for Public Health specialty training, 2018–2020 entry years.

The recruitment process has three stages: eligibility checking, the Assessment Centre and the Selection Centre (Fig. 1). On application, candidates are assessed to ensure they meet the baseline eligibility criteria set out in the person specification of either being a General Medical Council registered doctor who can demonstrate having achieved foundation programme competencies, or having a 2:1 undergraduate degree or a Masters degree and at least 48 months of work experience, 24 of which must be in a role relevant to population health practice.^2^ Eligible candidates are invited to the Assessment Centre, comprising three computer-based tests of numerical reasoning (RANRA), critical thinking (Watson Glaser Critical Thinking Assessment II, WGCTAII) and a bespoke situational judgement test (SJT). SJTs are widely used and are intended to quantify non-academic capabilities; they assess participants’ reactions to a range of hypothetical role-based scenarios.^3^ To be invited to Selection Centre, candidates must pass all three tests, and be ranked in the top 216 candidates. Between 2018 and 2020, the Selection Centre was held in person, and comprised a written exercise, a group exercise and six short ‘steeple-chase’ interviews. Candidates were deemed appointable if they met the pass mark (60%) for the Selection Centre. Appointable candidates were then ranked using a combination of Assessment and Selection Centre scores, and jobs allocated in rank order until all were filled.

Health Education England East Midlands (HEEEM), the lead recruitment organisation for UK Public Health specialty training, put in place measures to reduce discrimination in the recruitment process. Eligibility checkers were trained to assess consistently, and could not see candidates’ name, age or sex. The eligibility of candidates was assessed against the person specification^2^ by two people independently, and a process existed for resolving disagreements. The Assessment Centre used two well-established psychometric tests (RANRA and WGCTAII). The SJT was developed specifically for Public Health by occupational psychology experts, with input from subject matter experts, and piloted before use. At Selection Centre, all assessors had undertaken Equality and Diversity and Unconscious Bias training, and were not aware of candidates’ backgrounds. As far as possible, assessors were recruited from a diverse range of backgrounds, and each candidate was judged by at least 10 assessors.

However, these measures may not be sufficient to reduce differential attainment (DA). In recent years, interest in DA in medical specialties has grown. Doctors whose medical qualification was received outside the UK, and ethnic minority doctors, face increased barriers to recruitment into training,^4^ progression through training^5^^,^^6^ and recruitment to consultant posts.^7^^,^^8^

In 2020, the BMJ published a news story suggesting that for every specialty included, white candidates were more likely to be deemed appointable for specialty training than ethnic minority candidates.^9^ Among the specialties considered, Public Health had the greatest differential between white and ethnic minority candidates in the proportion deemed appointable. It is known that many psychometric tests show differences by age and ethnicity,^10^ and there is evidence of this occurring in the instruments used by the UK Public Health recruitment process.^11–13^ Although WGCTAII has been found to show negligible or small differences by sex, age and primary language, it shows large differences by ethnicity.^11^

Prompted by the BMJ article,^9^ this research was independently commissioned by the UK Public Health Recruitment Executive Group (a committee of Health Education England) to determine whether some groups of candidates for Public Health specialty training were less likely to be successful in their applications, and to determine at which stage(s) of the process this occurs. The analysis, drafting and decision to submit this paper were undertaken exclusively by the authors.

## Methods

### Data sources

Separate, partially redacted, datasets for those applying for places in 2018, 2019 and 2020 (*The years described relate to the August in which successful applicants would take up the role. The recruitment cycle begins the preceding November, such that for example those applying in November 2017 would take up post in August 2018, and for the purposes of this analysis are labelled as 2018.*) were retrieved from the application system (‘Oriel’) by HEEEM. These datasets covered applicant sex, age group, ethnicity, professional background and progression through the selection process. The files were passed to the research team, who cleaned, coded and collated them into a single 3-year dataset for analysis.

The unit of observation was application rather than unique candidate, as it was not possible to identify whether the same applicant applied more than once. Professional background was assumed based on the eligibility criteria of each candidate; however, we note that a small number of people with a primary medical qualification apply through the BOTM eligibility criteria as they are unable to fulfil the criteria for medical applicants.

### Data processing

Data processing and analysis were undertaken in STATA SE 17.0 for Mac. Three serial binary endpoint variables for progression were generated as: ‘passed Assessment Centre’ where a candidate achieved satisfactory scores in all three test components; ‘attended Selection Centre’ where a candidate ranked sufficiently highly to be eligible for interview; and ‘appointable’ where a candidate achieved the cut-off score for appointable candidates (60%) at Selection Centre. Denominators are presented based on the number of applicants eligible to progress at the respective stage. Due to the comparatively small number of black and ethnic minority candidates, ethnicity categories were aggregated upwards to create sufficiently large groups for analysis.

### Analytical approach

Descriptive analysis of application and progression was undertaken with counts and percentages. Logistic regression was used for both univariable and multivariable analysis producing odds ratios (OR) and adjusted odds ratios [with 95% confidence intervals (CI)], assuming two-tailed testing with an alpha of 0.05. Applicants missing demographic data are presented in descriptive analyses with count data but censored in regression analyses.

## Results

The combined dataset included 2430 applicants (Table 1) across the 3 years of recruitment. Of these, 178 (7.3%) voluntarily withdrew their application prior to eligibility checking, leaving 2252 applications for subsequent analyses. Of this group, 1432 (63.6%) of candidates were deemed eligible to proceed to Assessment Centre. Data were missing for ~2–3% (sex and age group) and 6% (ethnicity) of candidates. Professional background data were complete. ‘Pipeline’ progression diagrams are presented in the Supplementary Figures.

**Table 1:** Demographic, professional background and eligibility of applications for Public Health higher specialty training over 3 years (2018–2020 inclusive) (*N* = 2430)

	Application year 2018		Application year 2019		Application year 2020		Total applications made	Voluntarily withdrawn	Included in analyses	Eligible to proceed to AC	
	n	(%)	n	(%)	n	(%)	n	n	n	n	
Total [%]	732	[30.1]	769	[31.7]	929	[38.2]	2430	178 [7.3]	2252	1432	[63.6]
Sex [%] –Male –Female –Not disclosed	238 478 16	(32.5) (65.3) (2.2)	232 507 30	(30.2) (65.9) (3.9)	296 605 28	(31.9) (65.1) (3.0)	766 1590 74	65 [8.5] 107 [6.7] 6 [8.1]	701 1483	422 977 33	[60.2] [65.9] [48.5]
Age group [%] –≤29 –30-34 –35-39 –40-44 –45+ –Not disclosed	229 211 159 73 60 -	(31.3) (28.8) (21.7) (10.0) (8.2)	218 207 171 84 70 19	(28.3) (26.9) (22.2) (10.9) (9.1) (2.5)	261 275 184 101 77 31	(28.1) (28.5) (21.2) (10.6) (8.5) (2.1)	708 693 514 258 207 50	68 [9.6] 48 [6.9] 34 [6.6] 12 [4.7] 16 [7.7] –	640 645 480 246 191 50	399 423 310 152 123 25	[62.3] [65.6] [64.6] [61.8] [64.4] [50.0]
Ethnicity [%] –White British –White Other –Black –Asian –Mixed –Chinese –Other –Not disclosed	346 84 84 119 37 8 11 43	(47.3) (11.5) (11.5) (16.3) (5.1) (1.1) (1.5) (5.9)	390 66 80 113 25 16 19 50	(50.7) (8.6) (11.7) (14.7) (3.3) (2.1) (2.5) (6.5)	455 81 112 155 43 13 18 52	(49.0) (8.7) (12.1) (16.7) (4.6) (1.4) (1.9) (5.6)	1191 231 286 387 105 37 48 145	85 [7.1] 23 [10.0] 8 [2.8] 32 [8.3] 11 [10.5] 1 [2.7] 3 [6.2] 15 [10.3]	1106 208 278 355 94 36 45 130	796 123 131 197 60 27 27 71	[72.0] [59.1] [47.1] [55.5] [63.8] [75.0] [60.0] [54.6]
Professional background [%] –Medical –BOTM	328 404	(44.8) (55.2)	332 437	(43.2) (56.8)	378 551	(40.7) (59.3)	1392 1038	117 [11.3] 61 [4.4]	1331 921	336 484	[63.5] [63.6]

Application numbers trended upwards over the 3 years, with 732 applications in 2018 and 929 in the 2020 round (27% increase over 3 years). The demographic analysis demonstrates a preponderance of female applicants, at a ratio of ~2:1, which is consistent over time. A larger proportion of female applicants (65.9% compared with 60.2% for males) were deemed eligible to proceed to Assessment Centre. The cohort is similar in age structure year-on-year, with similar proportions of applicants being deemed eligible. Approximately half of applicants over the 3 years identified as white British (49.0%) with comparatively consistent proportions of Asian (15.9%, comprising Indian, Pakistani, Bangladeshi and any other Asian background) and black (11.4%, comprising black African, black Caribbean and any other black background) candidates contributing the two next largest ethnic groups. Progression to Assessment Centre ranged from 75.0% for the Chinese ethnic group (*n* = 37) to 47.1% for the black ethnic group (*n* = 286).

Although the number of applications from those with a medical background increased from 328 (2018) to 378 (2020), the proportion of medics decreased, with the BOTM group contributing 55.2% of applications in 2018 and 59.3% by 2020. Progression to Assessment Centre was almost identical between the two professional groups at ~64%, once candidates withdrawing from recruitment were factored in: but almost three times as many medics voluntarily withdrew (11.3 versus 4.4% for BOTM candidates).

Over the 3 years, 1432 candidates attended the Assessment Centre, of whom 710 (49.6%) scored sufficiently highly to ‘pass’ it (Table 2). Yet due to the limited number of Selection Centre places, only 601 (84.6%) ranked sufficiently highly to take-up their invitations to Selection Centre. Visualization of the recruitment ‘pipelines’ is provided by group (Supplementary, Fig. S1a–d).

**Table 2:** Progression from Assessment Centre testing for applications (2018–2020) by demographic and professional background: descriptive, univariable and multivariable^a^ analyses with count, percentage, odds ratio (OR), adjusted odds ratio (aOR) and respective 95% confidence intervals (CI)

	Attended AC	Successful in passing Assessment Centre			Ranked sufficiently highly and attended Selection Centre		
	*N*	*n*	OR (95% CI)	aOR (95 CI%)	*n*	OR (95% CI)	aOR (95 CI%)
Total	1432	710			601		
Sex							
- Male	422	209	Reference		177	Reference	
- Female	977	486	1.01 (0.80–1.27)	0.96 (0.74–1.25)	410	1.00 (0.79–1.26)	0.96 (0.74–1.26)
Age group in years							
- ≤ 29	399	263	Reference		241	Reference	
- 30–34	423	235	0.65 (0.49–0.86)	0.83 (0.60–1.15)	196	0.57 (0.43–0.75)	0.76 (0.55–1.05)
- 35–39	310	126	0.35 (0.26–0.48)	0.55 (0.38–0.79)	98	0.30 (0.22–0.41)	0.51 (0.36–0.73)
- 40–44	152	37	0.17 (0.11–0.25)	0.27 (0.17–0.43)	24	0.12 (0.08–0.20)	0.21 (0.13–0.36)
- 45+	123	35	0.21 (0.13–0.32)	0.31 (0.19–0.51)	28	0.19 (0.12–0.31)	0.33 (0.20–0.56)
Ethnicity							
White British	796	467	Reference		398	Reference	
White Other	123	69	0.90 (0.61–1.32)	0.85 (0.57–1.28)	60	0.95 (0.65–1.39)	0.92 (0.61–1.39)
Asian	131	63	0.33 (0.24–0.46)	0.24 (0.17–0.35)	51	0.35 (0.25–0.50)	0.24 (0.16–0.35)
Black	197	16	0.10 (0.05–0.17)	0.10 (0.06–0.17)	12	0.10 (0.05–1.19)	0.10 (0.05–0.19)
Chinese	27	16	1.02 (0.47–2.24)	0.62 (0.27–1.42)	14	1.08 (0.50–2.32)	0.59 (0.26–1.37)
Other	27	15	0.88 (0.41–1.91)	0.73 (0.32–1.65)	14	1.08 (0.50–2.32)	0.92 (0.40–2.10)
Mixed	33	33	0.86 (0.51–1.46)	0.75 (0.43–1.32)	26	0.76 (0.45–1.30)	0.64 (0.36–1.14)
Professional background							
Medical	585	372	Reference		345	Reference	
BOTM	847	338	0.38 (0.31–0.47)	0.40 (0.30–0.53)	256	0.30 (0.24–0.38)	0.31 (0.23–0.41)

^a^The multivariable regression model included all variables on the table.

Univariable logistic regression revealed no significant differences by sex for either endpoint. Those in the ≤ 29-year-old age band were more likely to progress than others, with some indication of age inversely correlating with progression. In the context of ethnicity, the black group was estimated to be 90% less likely to progress than white British candidates, and Asian candidates approximately two-thirds less likely to progress. The Chinese, white other and other ethnic groups did not return statistically significant results, but comprised much smaller numbers. There was lower progression for the BOTM professional group when compared with medics: BOTM candidates were ~60–70% less likely to progress from the Assessment Centre.

Multivariable regression adjusting for sex, age group, ethnicity and professional background did not materially influence the directions of the univariable associations. However, under the multivariable regression, the performance gap for older candidates increased slightly. Although the multivariable regression partially attenuated the performance gap for Asian candidates, the performance gap for black candidates persisted at 90%.

Over the 3 years of analysis, 601 (*Although there are 216 available places each year for the Selection Centre, a number of candidates cancel or fail to attend resulting in in ~600 candidates as opposed to the maximum of 648.*) candidates attended the Selection Centre of which 378 (62.9%) were deemed appointable (Table 3). At this point in the process, female candidates were more likely to be deemed appointable than male candidates (although this was not statistically significant). The oldest age group (45+ years) was the only sub-group in the analyses to show strongly statistically significant DA at this stage (OR) 0.22, 95% CI 0.09–0.53). There were no strongly statistically significant differences by ethnicity, although the sub-groups by this stage of recruitment were comparatively small. And although BOTM candidates were more likely to be deemed appointable than medical applicants, this difference was not statistically significant.

**Table 3:** Appointability of candidates completing Selection Centre: descriptive, univariable and multivariable analyses with count, percentage, odds ratio (OR), adjusted odds ratio (aOR) and respective 95% confidence intervals (CI)

	Attending SC *N*	Appointable *n*	Odds ratio (95 CI%)	Adjusted odds ratio (95 CI%)
Total	601	378		
Sex				
- Male	177	104	Reference	
- Female	410	266	1.30 (0.90–1.86)	1.18 (0.81–1.73)
Age group in years				
- ≤ 29	241	155	Reference	
- 30–34	196	131	1.11 (0.75–1.66)	1.08 (0.71–1.64)
- 35–39	98	60	0.88 (0.54–1.42)	0.74 (0.43–1.27)
- 40–44	24	16	1.11 (0.46–2.70)	0.98 (0.40–2.45)
- 45+	28	8	0.22 (0.09–0.53)	0.16 (0.06–0.40)
Ethnicity				
White British	398	266	Reference	
White Other	60	33	0.61 (0.35–1.05)	0.56 (0.32–0.99)
Asian	51	31	0.77 (0.42–1.40)	0.69 (0.37–1.27)
Black	12	6	0.49 (0.15–1.57)	0.39 (0.12–1.26)
Chinese	14	6	0.37 (0.13–1.09)	0.37 (0.12–1.10)
Other	14	8	0.66 (0.22–1.95)	0.59 (0.20–1.75)
Mixed	26	13	0.50 (0.22–1.10)	0.46 (0.21–1.03)
Professional background				
Medical	345	214	Reference	
BOTM	256	164	1.10 (0.78–1.53)	1.34 (0.90–1.99)

Additional descriptive analysis using disaggregated ethnicity categories showed that between 2018 and 2020, no candidates from black Caribbean (*n* = 24), mixed white and black African (*n* = 20), Bangladeshi (*n* = 17) or black other (*n* = 8) backgrounds were deemed appointable; this compares to 66.8% of white British candidates.

## Discussion

### Main findings of this study

This is the first study to explore DA and progression in recruitment to specialty training in Public Health in the UK. The analyses suggest that there is considerable variation in progression through the recruitment process, relating in particular to ethnicity, age and professional background. Perhaps surprisingly, the variation appears more related to the Assessment Centre stage of recruitment (involving standardized, computer-based aptitude testing, which is expected to reduce DA^14^), than the eligibility checking or Selection Centre (interview) stages. These findings suggest that structural inequalities in psychometric testing, already establishing in the literature,^10^ including for the tests used in this process,^11^ may be more a cause of DA than conscious or unconscious bias by interviewers. This confirms findings from other studies that DA occurs in machine marked medical school exams.^15^

Seventy per cent of successful candidates were female; as with other specialties^16^ this reflects the make-up of those applying, and we did not find significant DA by sex. Half of initial applicants identify as white British, suggesting that visibility of the scheme is high among those from ethnic minority backgrounds. The white ethnic group forms 78.4% of the population of England and Wales^17^ and 77.9% of the NHS workforce.^18^ The ethnic diversity of those applying to Public Health training is greater than either of those benchmarks, whereas the eventual combined cohort of white British and white other appointable applicants (79.1%) is broadly similar to them.

Therefore, there does appear to be DA when considering the higher levels of attrition among black and other minority ethnic groups through the process. This study finds clear evidence of so-far unexplained differential progression among black and Asian candidates that appears most evident at the Assessment Centre. Importantly, the multivariable regression shows there is residual DA when adjusting for other demographic and professional characteristics which had been hypothesized to be confounders. Only two aggregated ethnic groups occasionally outperformed the white British group—Chinese and other—and this was only at the Assessment Centre stage and the difference was not statistically significant. Among all of the analyses involving smaller ethnic groups, it is not possible to rule out Type I or (more likely) Type II error.

Two hypotheses seem most likely: that known properties of the aptitude tests are driving the differential,^10^ or that residual confounding is at play. In terms of the latter possibility, it may be that minoritized groups are less networked and therefore less able to benefit from encouragement and advice on how to prepare for the assessments. Other studies of progression in medical training have identified a lack of peer and supervisor networks as a cause of DA for ethnic minority doctors.^4^

Further analysis is urgently required in the Assessment Centre component to determine what is driving the effects observed. This could include using existing (e.g. UKMED for medical applicants) or new datasets to control for a wider range of demographic characteristics, including past educational attainment, first language and country of primary medical qualification.

That younger candidates performed better than older ones might be explained through bias of the tests themselves, or through inadvertent selection, whereby high capability candidates are recruited on first application, and lower performing candidates re-apply repeatedly (and will be older at each recruitment round). There is, however, evidence in the wider psychometric literature that suggests performance can diminish with age.^10^

The difference in performance between medical and BOTM candidates at Assessment Centre may be explicable in terms of their comparative homogeneity. By virtue of having completed medical school and foundation training, medical candidates are high achievers. In contrast, the BOTM eligibility criteria, namely an undergraduate degree graded 2:1 or above and 4 years of work experience, are less stringent, creating higher levels of heterogeneity. Medics may benefit from the SJT element of the Assessment Centre which is similar to an assessment undertaken earlier in their training, as well as, on average, more recent experience of test-taking in general. Conversely, BOTM applicants, while often 5 or 10 years since university exams, are thought to benefit from the more wide-ranging questioning at Selection Centre where their experience may confer an advantage.

Further analysis is required to confirm or reject these explanations. However, there are a number of modifications to the recruitment process which might reduce DA. These include the provision of more information about the process in advance, to reduce the effect of personal networks, additional support through the recruitment process for candidates from groups who appear to be disadvantaged by it and a thorough review of the whole process, including the tests used, to select instruments which do not show DA by age or ethnicity.

### What is already known on this topic

There is extensive evidence of DA by ethnicity in medical training in the UK.^19^ Analysis published in the news section of the BMJ in 2020 reported that for all specialties considered, white candidates were more likely to be deemed appointable than ethnic minority candidates.^9^ This is supported by studies outside medicine showing differences in invitation to interview^20–22^ and on psychometric tests by both age and ethnicity,^10^ with older and non-white candidates generally performing less well.

### What this study adds

This study is the first to provide a detailed analysis of success rates in recruitment into Public Health specialty training, covering age, ethnicity, sex and professional background on a multi-year basis. The sample size is large enough to identify differences between ethnic minority groups, demonstrating that black and Asian candidates are less likely to be successful than both white and other ethnic minority candidates. It is the first study to assess DA across each stage of the recruitment process, from eligibility checking to being deemed appointable, to determine where in the process particular groups are being disadvantaged.

The findings of this analysis are important for the Public Health community in the UK. The Faculty of Public Health has made clear its commitment to tackling inequalities of all kinds, both within the profession and across society. To be able to effectively serve all communities in the UK, the Public Health workforce, including the senior levels fed by the specialty training programme, needs to be representative of those communities.

### Limitations of this study

This study is large and comprehensive, examining a cohort of > 2000 candidates over 3 years of recruitment preceding the COVID-19 pandemic. There are comparatively few missing data and the patterns observed are mostly consistent.

There are limitations attributable to the granularity of the raw data, scope of data points collected and several assumptions implicit to the process. Chief among these is the statistical power to identify effects, which diminishes over the recruitment cycle as numbers reduce. It is possible that other non-white ethnic groups are disadvantaged, but numbers are insufficiently large for analysis. There are also potential overlaps among the professional group exposures, with some doctors applying through the BOTM route.

Residual confounding is also likely, potentially attributable to socioeconomic background or language. The extent to which SJTs are predicated on cultural understanding is not well understood, not least when the literature on their validity in medical recruitment is often structured around clinical issues (which is precluded in a mixed specialty).

## Conclusion

This study suggests that some groups are disadvantaged by the current system of recruitment into Public Health specialty training. There are some reasons for optimism that this analysis has identified: those from minoritized backgrounds are applying for specialty training in Public Health, the diversity of the workforce deemed appointable is similar to the benchmark population, and the Selection Centre does not appear to be a major driver of DA. Yet there are reasons for concern also: there is evidence of DA among black and Asian applicants, and older candidates, which at present is unexplained, although is likely to be caused in part by the psychometric instruments used. Further analysis of performance on separate elements of the Assessment Centre, re-analysis accounting for possible residual confounders and consideration of changes to the recruitment process to ensure fairness are urgently required.

## Supplementary data

Supplementary data are available at the *Journal of Public Health* online.

## Supplementary Material

DAmanuscript_JPH20221007_supplementary_fdac122

## Data Availability

The data underlying this article were provided by Health Education England by permission. Data will be shared on request to the corresponding author only with permission of Health Education England.

## Notes

**Fran Bury**, Specialty Registrar in Public Health and Honorary Research Fellow

**Mala Rao**, Senior Clinical Fellow

**Richard Pinder**, Clinical Senior Lecturer and guarantor
