# Supplementary material for: Differential attainment in public health specialty training recruitment in the United Kingdom: an observational analysis of applicants from 2018 to 2020
Source: J Public Health (Oxf). 2022 Nov 3;45(2):330–7. doi: 10.1093/pubmed/fdac122 (PMC10273379; doi:10.1093/pubmed/fdac122)
Supplement: DAmanuscript_JPH20221007_supplementary_fdac122 [file damanuscript_jph20221007_supplementary_fdac122.docx]

**Differential attainment in Public Health specialty training recruitment in the United Kingdom: an observational analysis of applicants from 2018 to 2020.**

Submission for the Journal of Public Health

**EMBARGOED – PLEASE CONFIRM WITH AUTHOR BEFORE RELEASE**

**Authors**

Fran Bury^1^ ORCID: 0000-0002-6305-1994

Mala Rao^1,2^ ORCID: 0000-0001-5504-6303

Richard Pinder^1^ ORCID: 0000-0002-7010-6009

**Affiliations**

1. Department of Primary Care and Public Health, School of Public Health, Imperial College London
2. Ethnicity and Health Unit, Department of Primary Care and Public Health, Imperial College London

**Key words**

**Public health, recruitment, medical training, ethnicity, differential attainment**

**Supplementary figures**

**
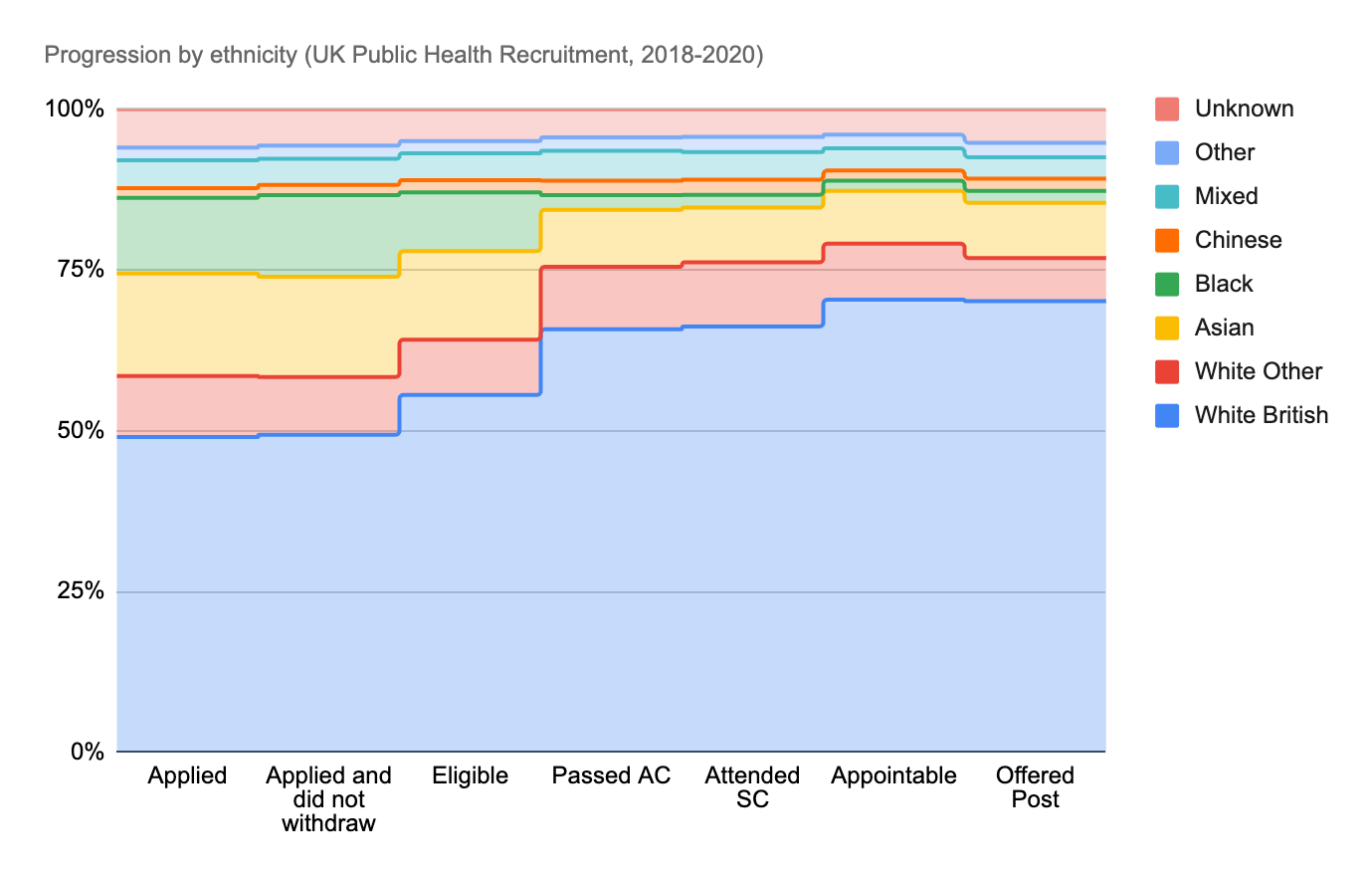
**

**Supplementary Figure 1a. Visualisation of progression pipeline by ethnicity, (UK Public Health Recruitment, 2018-20).**

**
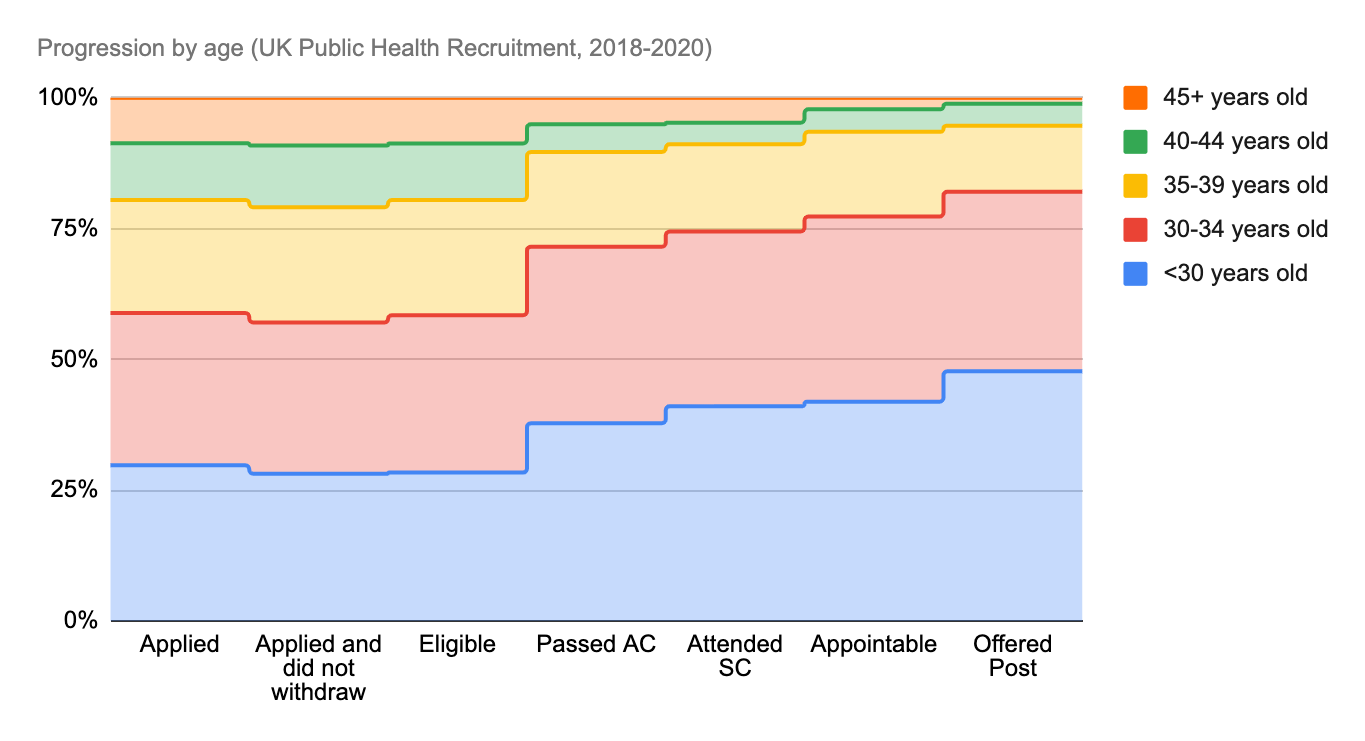
**

**Supplementary Figure 1b. Visualisation of progression pipeline by age, (UK Public Health Recruitment, 2018-20).**

**
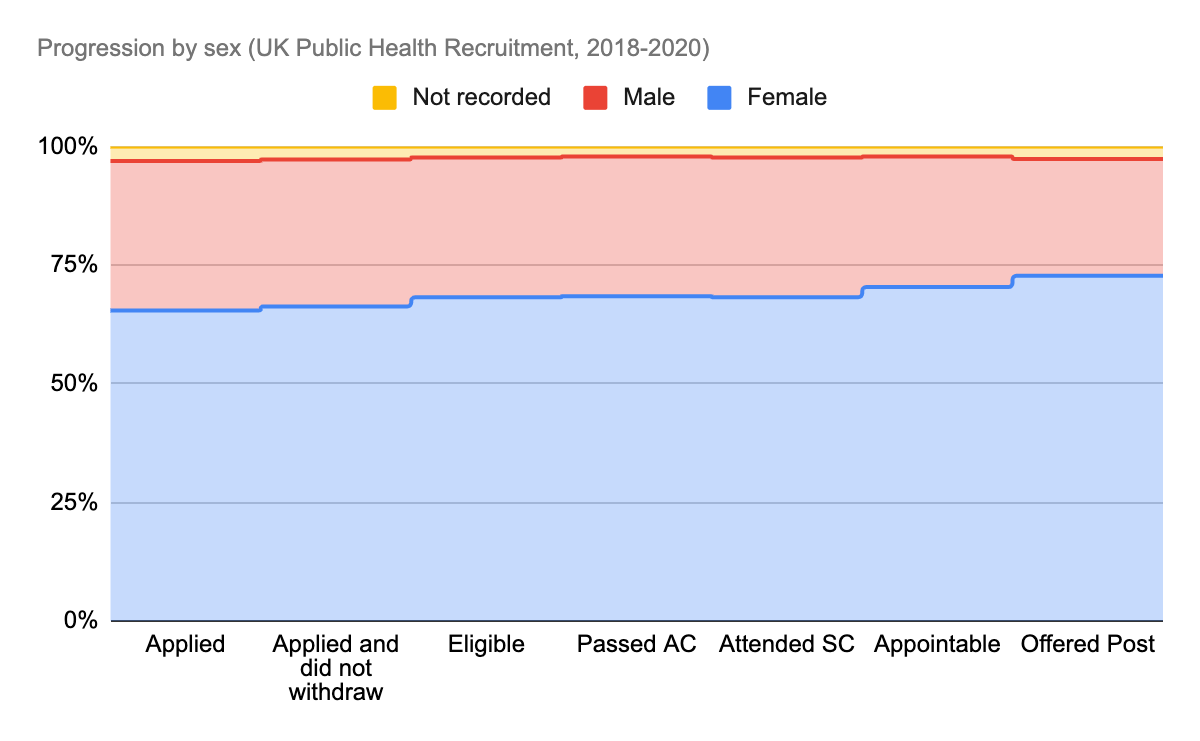
**

**Supplementary Figure 1c. Visualisation of progression pipeline by sex, (UK Public Health Recruitment, 2018-20).**

**
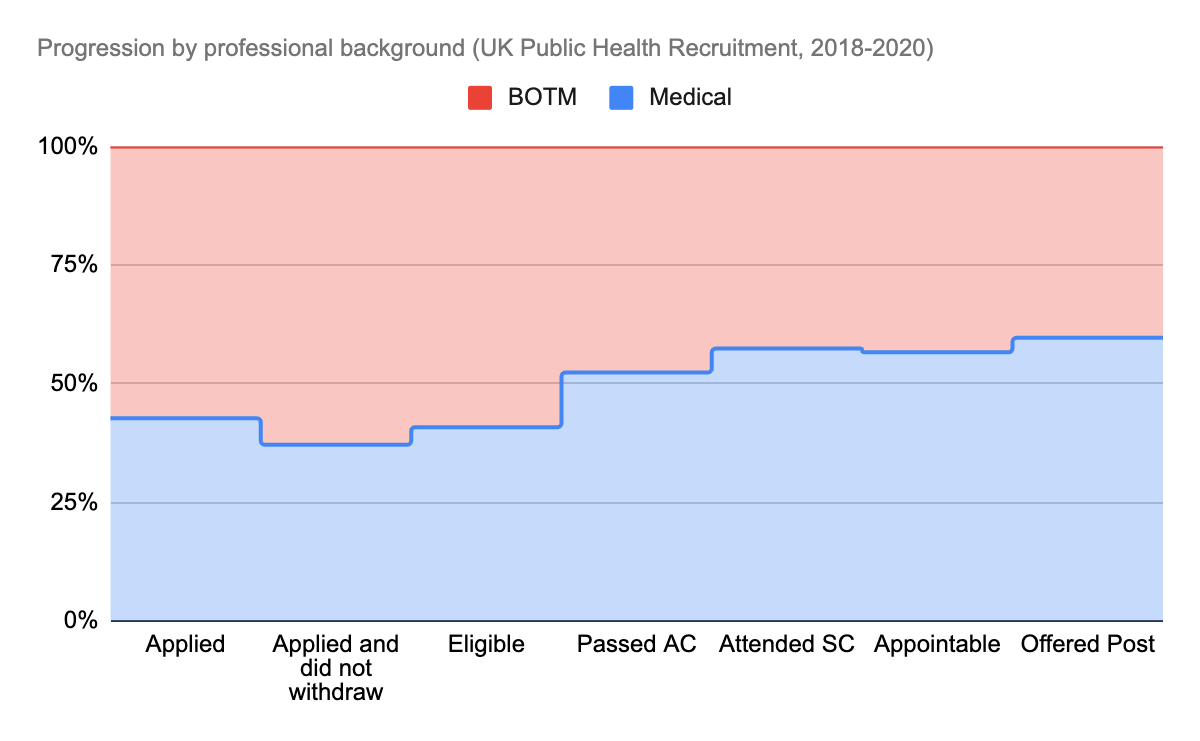
**

**Supplementary Figure 1d. Visualisation of progression pipeline by professional background, (UK Public Health Recruitment, 2018-20).**
